# Modulation of α-synuclein in vitro aggregation kinetics by its alternative splice isoforms

**DOI:** 10.1073/pnas.2313465121

**Published:** 2024-02-07

**Authors:** Alexander Röntgen, Zenon Toprakcioglu, James E. Tomkins, Michele Vendruscolo

**Affiliations:** ^a^Centre for Misfolding Diseases, Yusuf Hamied Department of Chemistry, University of Cambridge, Cambridge CB2 1EW, United Kingdom; ^b^Aligning Science Across Parkinson’s Collaborative Research Network, Chevy Chase, MD 20815

**Keywords:** alpha-synuclein, proteoforms, splice isoforms

## Abstract

The misfolding and aggregation of α-synuclein is linked to a family of neurodegenerative disorders known as synucleinopathies, the most prominent of which is Parkinson’s disease (PD). Understanding the aggregation process of α-synuclein from a mechanistic point of view is thus of key importance. *SNCA*, the gene encoding α-synuclein, comprises six exons and produces various isoforms through alternative splicing. The most abundant isoform is expressed as a 140-amino acid protein (αSyn-140), while three other isoforms, αSyn-126, αSyn-112, and αSyn-98, are generated by skipping exon 3, exon 5, or both exons, respectively. In this study, we performed a detailed biophysical characterization of the aggregation of these four isoforms. We found that αSyn-112 and αSyn-98 exhibit accelerated aggregation kinetics compared to αSyn-140 and form distinct aggregate morphologies, as observed by transmission electron microscopy. Moreover, we observed that the presence of relatively small amounts of αSyn-112 accelerates the aggregation of αSyn-140, significantly reducing the aggregation half-time. These results indicate a potential role of alternative splicing in the pathological aggregation of α-synuclein and provide insights into how this process could be associated with the development of synucleinopathies.

## Significance

The existence of proteoforms—distinct versions of a protein originating from the same gene via mechanisms such as genetic variation, RNA transcript alternative splicing, and post-translational modifications—considerably enriches the range of possible behaviors of that protein. Here, we investigated the impact of alternative splice isoforms of α-synuclein on its propensity to aggregate, a phenomenon closely associated with Parkinson’s disease and related synucleinopathies. Our observations reveal that even a relatively small amount of an isoform that is more prone to aggregation can hasten the overall aggregation of α-synuclein. These results illustrate the importance to factor in proteoforms when investigating protein behavior and their implications on disease pathology.

The aggregation of α-synuclein (αSyn) is central to the pathogenesis of Parkinson’s disease (PD) and other synucleinopathies, such as dementia with Lewy bodies (DLB) and multiple system atrophy (MSA) (1–4). αSyn is highly abundant in presynaptic terminals of neurons, where it is involved in several neuronal processes, such as synaptic transmission and plasticity, neuroprotection, and dopamine metabolism (2–4).

The amino acid sequence of the 140-residue isoform of αSyn (αSyn-140) can be divided into three functional regions—an amphipathic, lysine-rich N terminus (amino acids 1–60), a central hydrophobic non-amyloid-β component (NAC) region (amino acids 61–95), which is crucial for aggregation into cross–β amyloid fibrils, and a flexible, negatively charged C-terminus (amino acids 96–140) (5). However, it has become increasingly evident that the notion of proteins as single molecular species is incomplete and that a variety of proteoforms exists in cells, resulting in a complex modulation of protein behavior (6, 7). This is also the case for αSyn (8–13), prompting the question of whether different αSyn proteoforms could contribute to the association with disease of this protein. These proteoforms can be generated by post-translational modifications (PTMs), such as proteolytic truncation and chemical modification, as well as alternative splicing of αSyn RNA transcripts (9–11, 14, 15).

The *SNCA* gene, which encodes αSyn, consists of 6 exons, with exons 2–6 being protein-coding, and exon 1 as part of the 5′-untranslated region. By exon skipping, at least 3 more isoforms are produced: αSyn-126 (lacking exon 3, i.e., amino acids 41–54), αSyn-112 (lacking exon 5, i.e., amino acids 103–130), and αSyn-98 (lacking exons 3 and 5) (16–19). Despite extensive evidence that these three alternative splice isoforms are transcribed in human tissue (20), whether these transcripts are translated into proteins remains largely underexplored. This is in part due to technical constraints of conventional bottom-up proteomics approaches, however, with advances in top-down proteomics methods, intact full-length peptides can be detected (21). These methods have enabled the detection of αSyn-112 in brain tissue and blood (22, 23), providing initial evidence that at least this isoform is translated in vivo.

In its monomeric state, αSyn is intrinsically disordered, lacking a stable three-dimensional structure, which can be described in terms of conformational ensembles (24–27). The aggregation process of αSyn consists of several microscopic steps (28–30). Upon primary nucleation, αSyn monomers assemble into disordered oligomers, which in turn convert into β-sheet-rich fibril seeds. By further monomer addition, these seeds elongate into protofilaments and eventually mature amyloid fibrils. Secondary processes such as fibril fragmentation and, under certain conditions, fibril surface-catalyzed secondary nucleation increase the number of elongation-competent species, thus contributing to the overall aggregation (28, 29). Previously, the deletion of exon 5 has been reported to increase the aggregation propensity of αSyn due to the loss of parts of the negatively charged C-terminus (8, 19).

In this study, we performed a kinetic analysis of the effects of exon deletions on the aggregation of αSyn and investigated the effects of the shorter αSyn isoforms in their monomeric and fibrillar forms on the aggregation of the main isoform, αSyn-140. We further performed a structural characterization of the aggregates by transmission electron microscopy (TEM) and an assessment of neuroblastoma cell viability upon exposure to the different isoform aggregates. Our findings underscore the significance of considering proteoforms in studies of αSyn activity and their consequent effects on disease development and progression.

## Results

### αSyn-112 and αSyn-98 Aggregate Faster than αSyn-140 and αSyn-126.

We initially used the CamSol method (31, 32) to predict the effect of exon deletions on the solubility and aggregation of αSyn. This analysis indicated that the deletion of exon 5 should reduce the solubility of αSyn, whereas the deletion of exon 3 should have essentially no effect (*SI Appendix*, Fig. S1). αSyn isoforms were then expressed recombinantly and obtained at high purity (*SI Appendix*, Fig. S2).

**Fig. 1.**
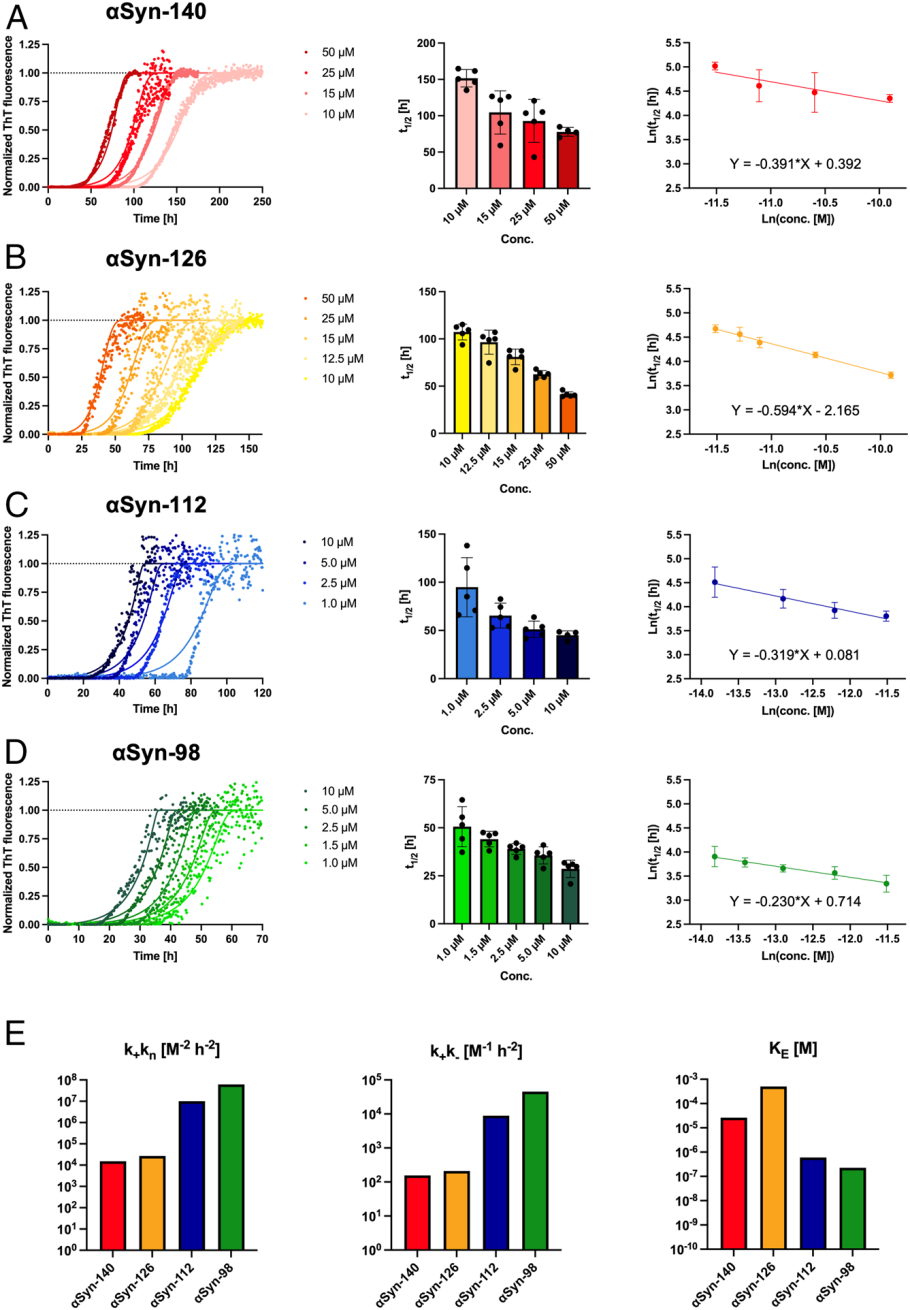
Aggregation kinetics of the αSyn isoforms. (*A*–*D*) The aggregation of the αSyn isoforms was assessed by measuring ThT fluorescence intensity over time. Normalized traces (*Left* panels) were fitted using AmyloFit with the *Saturating Elongation and Fragmentation* model, shown as the solid lines. The aggregation half-times (t_1/2_) exhibit a clear concentration dependence (*Central* panels), and double-logarithmic plots (*Right* panels) were used to determine the scaling exponent (i.e., the slope of linear regression function) for selection of the appropriate aggregation model (33). Values of t_1/2_ are shown as mean ± SD. (*E*) Kinetic parameters derived from the fitting are k_+_k_n_ (primary nucleation and elongation), k_+_k_–_ (elongation and fragmentation), K_E_ (concentration of half-maximal elongation speed) (33).

We next investigated the aggregation kinetics of the αSyn isoforms at different concentrations by monitoring the increase in ThT fluorescence intensity over time (*Materials and Methods*). αSyn-140 and αSyn-126 incubated at 10 to 50 µM (Fig. 1 *A* and *B*) were found to aggregate on longer timescales than αSyn-112 and αSyn-98 incubated at 1 to 10 µM (Fig. 1 *C* and *D*). Using AmyloFit (33), we determined the scaling exponent and the model of aggregation (*Saturating Elongation and Fragmentation*) for the best fit of the data. With this approach, we derived the microscopic rate constants for the aggregation process (Fig. 1*E* and *SI Appendix*, Table S1). For this, the reaction order of primary nucleation n_c_ was set to 2 in all cases. The combined rate constant k_+_k_n_, which describes primary nucleation and fibril elongation, was increased by three orders of magnitude for αSyn-112 and αSyn-98 compared to αSyn-140 and αSyn-126, similar to the parameter k_+_k_–,_ which corresponds to fibril elongation and fragmentation. In contrast, αSyn-112 and αSyn-98 reached their half-maximal fibril elongation speed at two to three orders of magnitude lower concentrations than αSyn-140 and αSyn-126, respectively, which is reflected by parameter K_E_. These data clearly demonstrate that deletion of exon 5, as in αSyn-112 and αSyn-98, drastically accelerates aggregation, whereas deletion of exon 3, as in αSyn-126, has little effect on the aggregation properties. αSyn-98 containing deletions of both exons appears to aggregate fastest, although deletion of exon 5 seems to account for a greater proportion of this acceleration than deletion of exon 3, comparing αSyn-98 to αSyn-126 and αSyn-112, respectively. For comparison, we also report fits using two alternative models (*SI Appendix*, Fig. S3), which deviate more strongly from the data and therefore do not appear suitable compared to the model selected here.

### Sequence Deletions in αSyn Isoforms Promote the Formation of Distinct Amyloid Polymorphs.

**Fig. 2.**
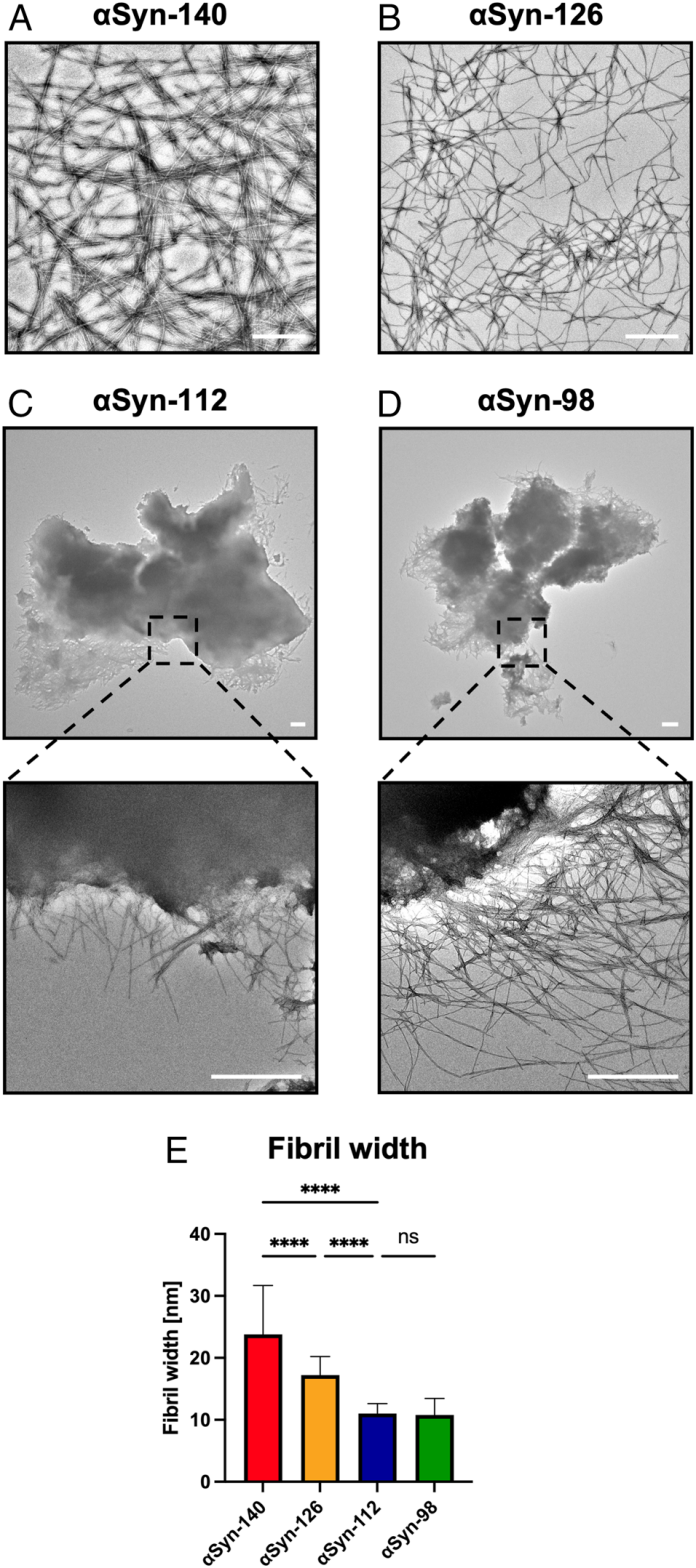
αSyn isoforms form morphologically distinct aggregates. (*A*–*D*) Representative TEM images of αSyn isoform aggregates. αSyn-140 and αSyn-126 formed elongated amyloid fibrils (*A* and *B*), whereas αSyn-112 and αSyn-98 formed clumps of several microns in diameter comprised of a fibrillar network (*C* and *D*). Fibrils are visible radiating out of these clumps, as shown in the representative images at higher magnification. (*E*) Image analysis indicating differences in fibril widths between the αSyn isoforms. Data are shown as mean ± SD. One-way ANOVA with Tukey’s post-hoc test. ****P < 0.0001, ns = non-significant. (Scale bars, 1 µm.)

Having established that variations in protein sequence affect aggregation kinetics, we next investigated whether these variations also correlate with different aggregate morphologies. By employing the use of transmission electron microscopy (TEM), we found that αSyn-140 and αSyn-126 aggregated into elongated amyloid fibrils (Fig. 2 *A* and *B*). In contrast, both αSyn-112 and αSyn-98 were found to form amyloid fibrils that assembled into few distinct clumps, several microns in diameter. The clumps consisted of a network of amyloid fibrils, and fibrils were visualized radiating out at the edge of these clumps (Fig. 2 *C* and *D*). Furthermore, fibrils of αSyn-126 had significantly smaller widths than αSyn-140 fibrils. αSyn-112 and αSyn-98 fibrils had similar widths compared to each other but had in turn significantly smaller widths than αSyn-126 fibrils (Fig. 2*E*). Thus, deletion of exon 5 not only accelerates aggregation but it also induces the formation of a distinct type of aggregates consisting of clumped amyloid fibrils.

### αSyn-112 Accelerates the Aggregation of αSyn-140.

**Fig. 3.**
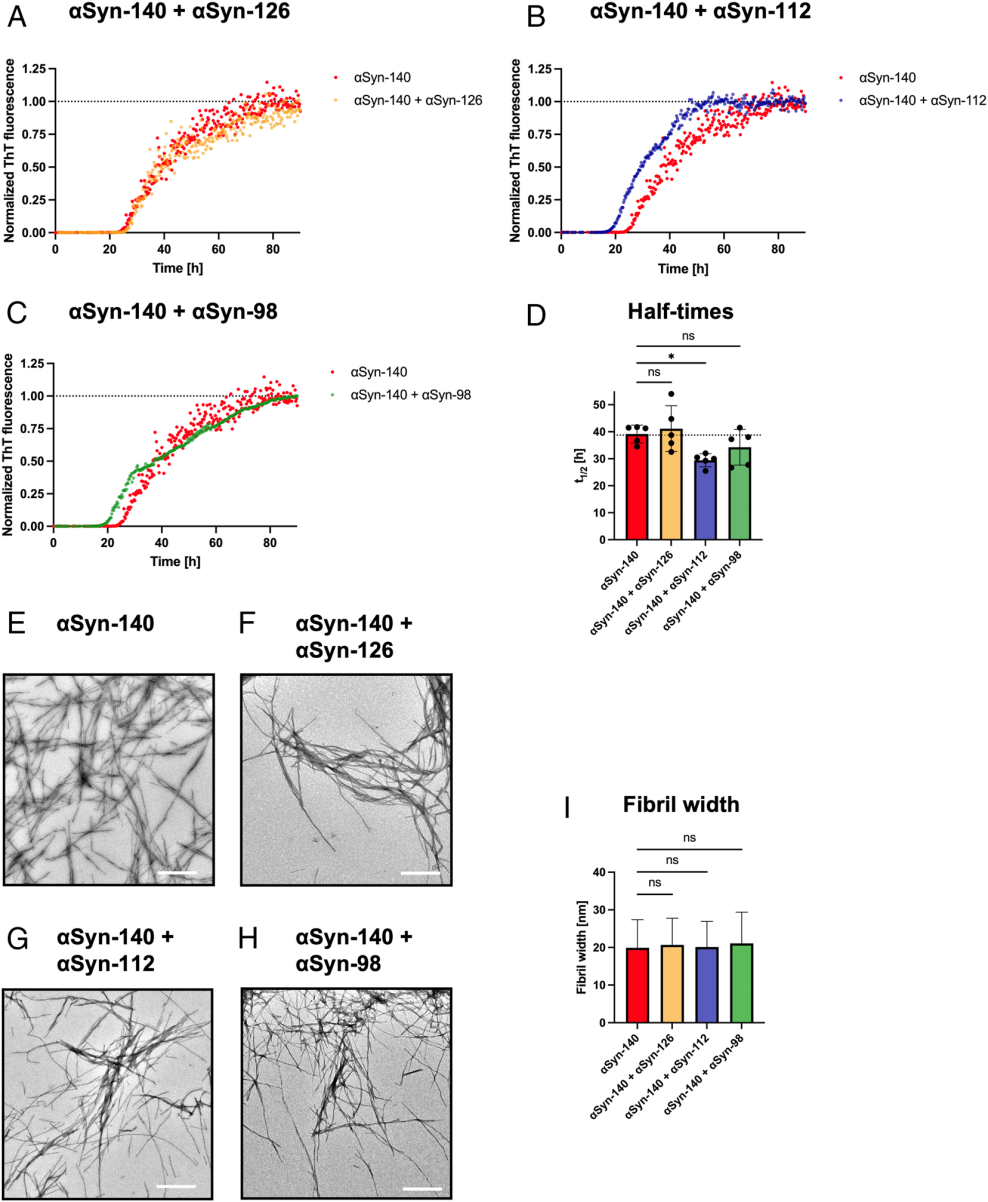
Co-aggregation assays of αSyn isoforms. (*A*–*C*) Aggregation of 100% (50 µM) αSyn-140 was compared with mixtures of 90% (45 µM) αSyn-140 with 10% (5 µM) αSyn-126, αSyn-112 or αSyn-98 monomers by monitoring the increase in ThT fluorescence over time. (*D*) Half-time analysis revealed a significant acceleration of αSyn-140 aggregation by ~25% upon co-incubation with 10% αSyn-112, but no difference when co-aggregated with 10% αSyn-126 or αSyn-98. (*E*–*I*) Representative TEM images of amyloid fibrils formed by αSyn-140 and αSyn isoform mixtures (*E*–*H*), which possess similar fibril widths (*I*). Data are shown as mean ± SD. One-way ANOVA using Dunnett’s post hoc test. *P < 0.05, ns = non-significant. (Scale bars, 1 µm.)

Having elucidated the effect of exon deletions in αSyn isoforms on their individual aggregation behavior, we further analyzed whether αSyn isoforms can modulate the aggregation of αSyn-140. Since αSyn-140 is estimated to be expressed at higher levels in the brain than αSyn-126, αSyn-112, and αSyn-98 (14, 34, 35), we chose a ratio of 9:1 for monomer co-aggregation experiments. Of the three alternative isoforms, only αSyn-112 led to a significant acceleration of aggregation kinetics, as indicated by a decrease in t_1/2_ values by ~25% (Fig. 3 *B* and *D*). In contrast, no significant effect was observed by addition of αSyn-126 or αSyn-98, whereas the latter exhibited biphasic aggregation (Fig. 3 *A*, *C*, and *D*). Moreover, TEM analysis revealed that all monomer mixtures formed elongated amyloid fibrils with similar widths as observed for the αSyn-140 control, while no clumps were observed for mixtures containing αSyn-112 and αSyn-98 (Fig. 3 *E*–*I*), as was the case for aggregates formed by these isoforms alone (Fig. 2 *C* and *D*). It could be possible that αSyn isoform monomers were incorporated into αSyn-140 fibrils without significantly affecting the morphology.

### αSyn Isoform Fibrils Have a Reduced Capacity to Seed αSyn-140 Aggregation but Affect the Aggregate Morphology.

**Fig. 4.**
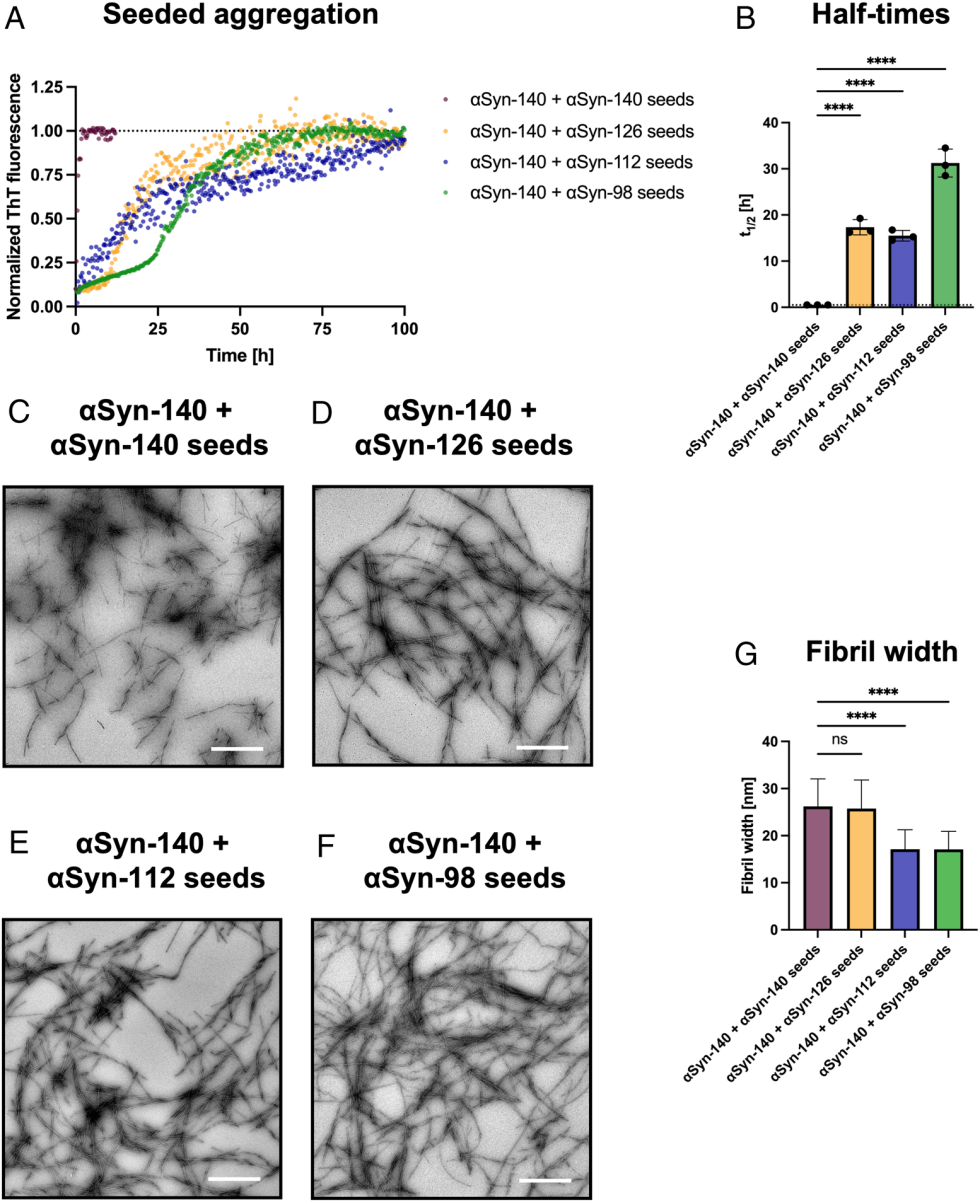
Seeded aggregation assays of αSyn-140. (*A*) Normalized aggregation kinetics of 90% (45 µM) αSyn-140 co-incubated with 10% (5 µM) fibril seeds of αSyn-140 (= self-seeded), αSyn-126, αSyn-112, or αSyn-98 (= cross-seeded). (*B*) While the αSyn-140 self-seeded reaction reached plateau rapidly, t_1/2_ values of cross-seeded reactions were significantly increased for αSyn-126 seeds and αSyn-112 seeds and highest for αSyn-98 seeds. (*C*–*F*) Representative TEM images of amyloid fibrils formed by αSyn-140 seeded aggregation. (*G*) Image analysis revealed significantly lower fibril widths of αSyn-140 cross-seeded with αSyn-112 and αSyn-98 compared to αSyn-140 seeded with αSyn-140 and αSyn-126. Data are shown as mean ± SD. One-way ANOVA using Dunnett’s post hoc test. ****P < 0.0001, ns = non-significant. (Scale bars, 1 µm.)

Given the faster aggregation of αSyn-112 and αSyn-98 compared to αSyn-140 monomers (Fig. 1) and that the aggregation of αSyn-140 was significantly accelerated by co-aggregation with αSyn-112 monomers (Fig. 3 *B* and *D*), we complemented the analysis by investigating the effect of pre-formed fibril seeds of the alternative αSyn isoforms on αSyn-140 monomer aggregation (Fig. 4). This process is also referred to as cross-seeding (i.e., addition of fibril seeds of a different isoform) in contrast to self-seeding (i.e., addition of fibril seeds of the same isoform). We chose a fibril concentration of 10% monomer equivalents, equal to that of αSyn isoform monomers during the co-aggregation experiments (Fig. 3). At such high fibril seed concentrations, primary nucleation is by-passed (36, 37), and fibril elongation becomes the dominant process in self-seeded experiments. As expected, αSyn-140 self-seeding was most efficient, leading to an instantaneous increase in ThT fluorescence intensity. However, the aggregation was significantly delayed when αSyn-140 was cross-seeded with αSyn-126 and αSyn-112. These results can be explained by the impaired structural compatibility of the fibril seeds with αSyn-140 monomers, as both isoforms lack the protein sequence encoded by one exon. Consequently, cross-seeding with αSyn-98 was the least efficient, lacking the protein sequence encoded by two exons, with a t_1/2_ value similar to that of the αSyn-140 unseeded control (*SI Appendix*, Fig. S4). Elongated amyloid fibrils were formed in all conditions as assessed by TEM. However, αSyn-140 cross-seeded with αSyn-112 and αSyn-98 exhibited significantly reduced fibril widths compared to αSyn-140 self-seeded and unseeded controls as well as those cross-seeded with αSyn-126 (Fig. 4*G* and *SI Appendix*, Fig. S4). All aggregates formed in this study were further analyzed by Fourier-transform infrared (FTIR) spectroscopy (*SI Appendix*, Fig. S5). While slight shifts in the amide I and II bands were observed for aggregates of the pure isoforms (*SI Appendix*, Fig. S5*A*), no significant differences were observed for co-aggregated or cross-seeded fibrils (*SI Appendix*, Fig. S5 *B* and *C*), indicating that the αSyn fibrils contain a similar secondary structure content.

### Aggregates of Different αSyn Isoforms Exhibit Different Effects on Cell Viability.

**Fig. 5.**
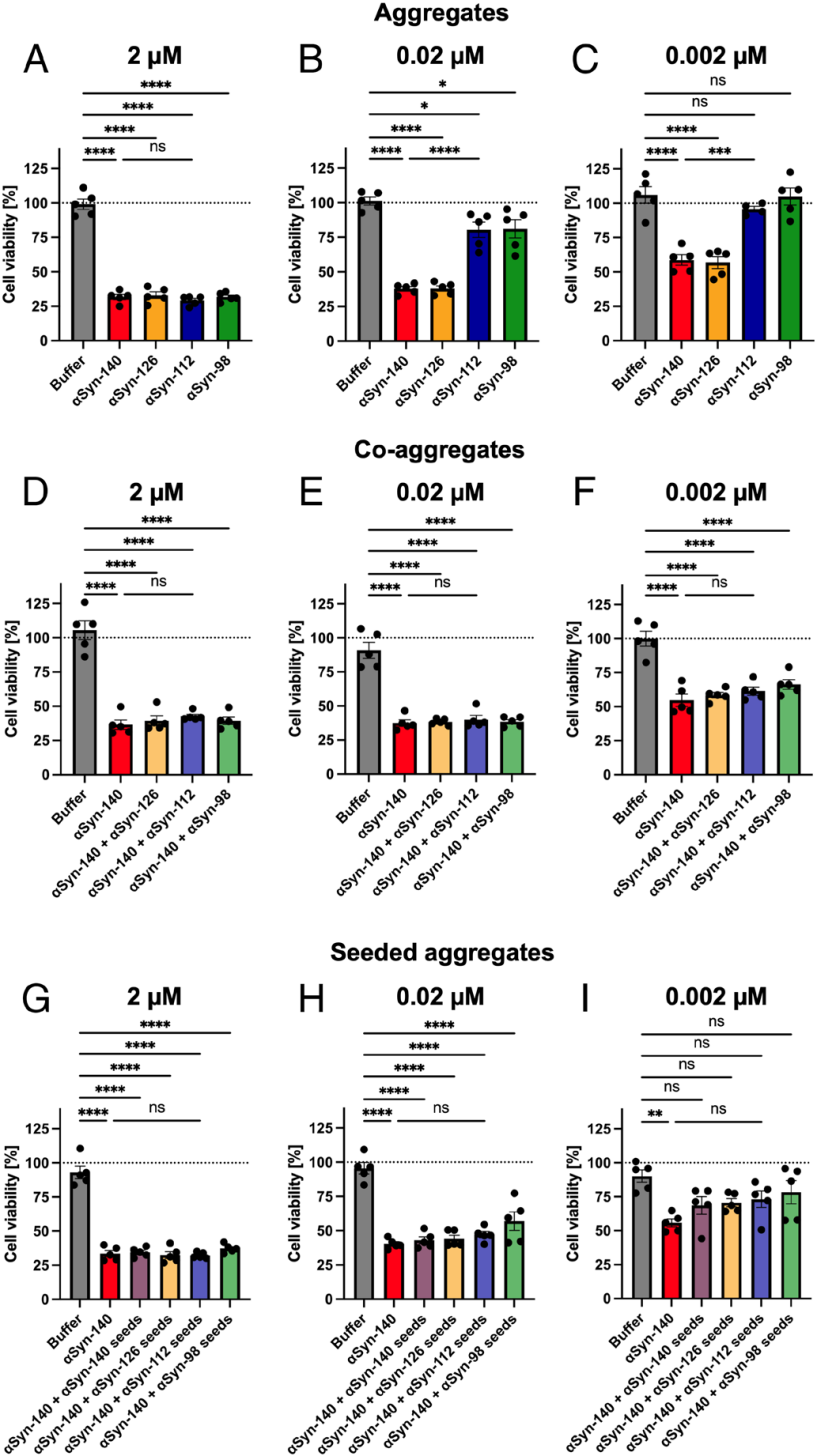
Effects of αSyn isoform aggregates on cell viability. (*A*–*I*) Cell viability of SH-SY5Y cells was determined using an MTT assay after treatment with fibrils formed by aggregation of 100% αSyn-140, αSyn-126, αSyn-112, and αSyn-98 (*A*–*C*), co-aggregation of 90% αSyn-140 with 10% αSyn-126, αSyn-112, or αSyn-98 monomers compared to 100% αSyn-140 (*D*–*F*), and seeded aggregation of 90% αSyn-140 monomers with 10% αSyn-140, αSyn-126, αSyn-112, or αSyn-98 fibril seeds (*G*–*I*); the results are compared to 100% αSyn-140, at 2 µM (*A*, *D*, and *G*), 0.02 µM (*B*, *E*, and *H*) and 0.002 µM (*C*, *F*, and *I*) final concentrations (monomer equivalents). Data are expressed as percentage of medium control and shown as mean ± SEM of independent cell treatments (n = 5). One-way ANOVA with Tukey’s post hoc test, ****P < 0.0001, ***P < 0.001, **P < 0.01, *P < 0.05, ns = non-significant.

Aggregates of αSyn, both in oligomeric and fibrillar forms, have previously been shown to possess cytotoxic properties, which are thought to be a crucial factor for neurodegeneration in PD and other synucleinopathies (38–41). Therefore, we assessed the effects of αSyn isoforms on SH-SY5Y neuroblastoma cells using an MTT cell viability assay (*Materials and Methods*). First, we confirmed that monomers of all αSyn isoforms exhibited no effects even at an elevated concentration (50 µM) (*SI Appendix*, Fig. S6). We then measured the effects of αSyn aggregates starting with a concentration of 2 µM monomer equivalents (Fig. 5). At this concentration, cell viability was reduced to ~30% upon treatment with aggregates of all isoforms (Fig. 5*A*). In order to investigate whether the observed reduction in cell viability was a dose-dependent effect, we gradually decreased the aggregate concentration. We found that cell viability was reduced less at a protein concentration of 0.02 µM and 0.002 µM compared to 2 µM, and aggregates of αSyn-112 and αSyn-98 had significantly less effects than αSyn-140 and αSyn-126 (Fig. 5 *B* and *C*). In addition, we assessed the effects of aggregates formed via co-incubating 90% αSyn-140 with 10% αSyn isoform monomers (Fig. 5 *D*–*F*). Moreover, we investigated the effects of cross-seeded aggregation with 10% isoform fibril seeds (Fig. 5 *G*–*I*). Fibrils from monomer co-aggregation and cross-seeded reactions showed similar effects compared to the αSyn-140 control, at all concentrations tested. This is in line with the fact that the aggregation reaction for these conditions mainly consisted of αSyn-140.

## Discussion

We have reported a detailed biophysical characterization of three αSyn splice isoforms—αSyn-126, αSyn-112, and αSyn-98—and of their interaction with the main αSyn-140 isoform. We have shown that deletion of the C-terminal sequence encoded by exon 5 significantly enhances αSyn aggregation (Fig. 1), as expected from solubility predictions based on the physicochemical properties of the amino acid sequences of the four isoforms (*SI Appendix*, Fig. S1). These findings can be rationalized by the high negative charge of the C-terminal region of αSyn-140, which is protective against aggregation. Previous studies on C-terminally truncated αSyn variants also reported an increased aggregation propensity compared to αSyn-140 (42–47), while reports using end-point aggregation assays in cultured cells have either indicated an increased or decreased aggregation propensity for αSyn-112 and αSyn-98 (35, 48, 49).

In our experimental setup, we did not observe major differences in aggregation upon deletion of the N-terminal exon 3, comparing αSyn-140 with αSyn-126, and αSyn-112 with αSyn-98. However, the N-terminal sequence forms part of a region containing seven highly conserved 11-amino acid repeats that adopts an α-helical structure upon lipid binding (50–52), which facilitates primary nucleation (29). Familial mutations located in this region have been shown to drastically affect lipid-induced aggregation (53). It is therefore possible that deletion of exon 3 modulates the aggregation propensity of αSyn in the presence of lipids in vitro or in cellular systems.

Furthermore, deletion of exon 5, as in αSyn-112 and αSyn-98, greatly affected aggregate morphology, inducing the formation of dense fibril clumps, while leading to a decrease in fibril width (Fig. 2). This result is in line with studies showing that truncations or familial point mutations affect αSyn fibril structure (47, 54) but contrasts the report that αSyn-98 formed small circular assemblies (35). However, in this regard, it should be noted that amyloid structures are not only highly sensitive to changes in the corresponding amino acid sequence but also to the specific assay conditions used to generate them, including protein concentration, pH, ionic strength, temperature, and agitation (55).

Besides the differences in aggregation propensity of the individual isoforms, we found that αSyn-112 accelerates the aggregation of αSyn-140 (Fig. 3). This result indicates that changes in alternative splicing may play a role in the development of synucleinopathies and is consistent with reports that alternative αSyn transcripts are found at different levels in diseased brains (8, 18–20, 34, 56–58). Specifically, αSyn-112 expression was found to be increased in PD cerebellum (58), DLB frontal cortex (34, 56), and MSA substantia nigra, striatum, cerebellar cortex, and prefrontal cortex (59). Moreover, αSyn-112 expression was increased relative to total αSyn in frontal cortex of individuals with PD risk alleles in the 3′ region of the αSyn transcript (60).

We complemented the kinetic analysis by cross-seeding experiments, showing that exon deletion gradually reduces the capacity of fibrils to seed the aggregation of αSyn-140 monomers (Fig. 4). Similarly, decreased cross-seeding capacity had also been previously shown for truncated αSyn variants (46). Therefore, it may be plausible for enhanced primary nucleation between αSyn-112 and αSyn-140 monomers, rather than cross-seeding by αSyn-112 fibrils formed in situ, to underlie the observed acceleration upon monomer co-aggregation. Interestingly, using αSyn-112 and αSyn-98 fibrils, which possessed smaller widths, to cross-seed αSyn-140 monomers in turn resulted in a smaller fibril width, indicating that the templating by fibril seeds could influence the final morphology.

Lastly, we carried out cell studies that indicated that αSyn-112 and αSyn-98 aggregates have less effects on cell viability than αSyn-140 and αSyn-126 aggregates (Fig. 5). This finding may be due to increased sequestration of fibrils in clumps, which are therefore less available for interaction with cells. In contrast, a recent study has demonstrated that intracellular overexpression of αSyn-112 and αSyn-98 led to greater cytotoxicity than αSyn-140 and αSyn-126, underlining a pathogenic potential of these isoforms (49). In our system, fibrils from co-aggregation and cross-seeding experiments had similar effects on cell viability as fibrils from pure αSyn-140 aggregation.

## Conclusions

This study has provided a characterization of the aggregation of αSyn splice isoforms, reporting evidence that αSyn-112, even at low levels, enhances the aggregation of αSyn-140. Based on these results, we anticipate that the detection of αSyn splice isoforms at the protein level using antibody or proteomic approaches will be of value to further understand the contributions of alternative splice variants in the pathogenesis of synucleinopathies. Furthermore, future experiments using in vivo models of αSyn aggregation could provide valuable insights into the contributions of αSyn isoforms to the association of this protein with disease.

## Materials and Methods

### Recombinant Production of αSyn Isoforms.

αSyn-140 was expressed from the pT7-7 plasmid. Plasmids for expression of αSyn-126, αSyn-112, and αSyn-98 were obtained from GenScript Biotech. The DNA sequences, lacking exon 3, exon 5, or both, respectively, were cloned into the NdeI/XhoI restriction sites of pET29a(+) plasmids and transformed into BL21-Gold (DE3) *Escherichia coli* (Agilent Technologies, #230132). αSyn isoforms were then recombinantly produced and purified based on previously described protocols (46, 61, 62). All isoforms were finally purified by size exclusion chromatography using 50 mM Tris-HCl, pH 7.4, snap-frozen in liquid N_2_, and stored at –80 °C until further use.

### Aggregation Assays.

For assessment of protein aggregation, αSyn isoforms were diluted to the chosen concentrations in 50 mM Tris-HCl, pH 7.4, with 50 µM thioflavin T (ThT) (33). The solutions were transferred to a Corning^®^ 96-well Half-Area Black with Clear Flat Bottom Polystyrene Non-Binding Surface Microplate at 100 µL per well, containing a 3-mm borosilicate bead (Merck). Plates were sealed using Thermowell^TM^ Sealing Tape (Corning) to avoid evaporation and incubated on a FLUOStar Omega plate reader (BMG Labtech) at 37 °C under quiescent conditions. For seeded aggregation, αSyn fibrils were recovered from plates after 6 d (from 50 µM for αSyn-140, αSyn-126, coaggregation and seeded reactions, and 10 µM for αSyn-112, αSyn-98 aggregation reactions). Fibril concentrations (in monomer equivalents) were calculated based on full conversion of monomers. Fibrils were sonicated at 10 µM in 300 µL total volume using a Sonopuls HD2070 microtip sonicator (Bandelin). Then, fibril seeds were added to αSyn monomers and aggregation assays were performed as described above for unseeded assays.

### Kinetic Analysis.

The kinetic data obtained from the aggregation assays were normalized with respect to minimum and maximum ThT fluorescence intensities. Half-times (t_1/2_) were calculated based on the time when fluorescence intensity reached 50% of its maximum value. All experiments were performed with three or five repeats, median traces were chosen for representation and, if required, further analyzed using the AmyloFit 2.0 platform (https://amylofit.com/) to derive microscopic rate constants (33).

### Transmission Electron Microscopy.

Samples for transmission electron microscopy (TEM) were recovered from aggregation reactions and prepared on carbon films on 3-mm 300-mesh copper grids (EM Resolutions Ltd.) (DOI: dx.doi.org/10.17504/protocols.io.q26g7pd79gwz/v1). For this, 2.3 μL of sample was spotted. The sample was then stained with 2.3 μL of 2% (w/v) uranyl acetate. TEM images were acquired on an FEI Talos F200X G2 transmission electron microscope. Fibril widths were quantified using ImageJ 1.53k (https://imagej.net/, RRID:SCR_003070) measuring at least 200 fibrils from at least eight different images per condition.

### Cell Culture.

Human SH-SY5Y neuroblastoma cells (RRID:CVCL_0019) were cultured in DMEM/F-12, GlutaMAX™ (ThermoFisher Scientific) supplemented with 10% (v/v) fetal bovine serum (FBS) on 75 cm^2^ treated polystyrene flasks (Greiner Bio-One) (DOI: dx.doi.org/10.17504/protocols.io.36wgq3bk5lk5/v1). Cells were grown at 37 °C in a 5% CO_2_-humified atmosphere and split at 80% confluency. The cell line was authenticated using short tandem repeat (STR) analysis by the Cancer Research UK Institute, and the cells tested negative for *mycoplasma* contaminations.

### MTT Cell Viability Assay.

Cell viability was measured using the 3-(4,5-dimethylthiazol-2-yl)-2,5-diphenyltetrazolium bromide (MTT) assay (DOI: dx.doi.org/10.17504/protocols.io.e6nvwdoqwlmk/v1). For application to cells, αSyn aggregates were prepared under similar conditions as for kinetic experiments and recovered after 6 d of incubation. However, no ThT was used in these experiments, and seeds were prepared by sonication in a closed Eppendorf tube in an Ultrawave Ultra BT U100 water bath sonicator for 10 min.

Cells were seeded on 96-well plates (Greiner Bio-One) at a density of 10,000 cells/well in 100 µL medium. After incubation for 24 h at 37 °C, the medium was discarded and replaced either with fresh medium (medium control), 20% (v/v) 50 mM Tris-HCl, pH 7.4, in fresh medium (buffer control), or αSyn aggregates at 20% (v/v) 50 mM Tris-HCl, pH 7.4, in fresh medium. Cells were treated in quintuplicates and incubated for another 24 h at 37 °C. Medium was discarded, and cells were incubated with 0.5 mg/mL MTT in RPMI medium (ThermoFisher Scientific) for 4 h at 37 °C. The solution was discarded, and the formazan product was solubilized by incubation at 500 rpm and 37 °C for 15 min in 100 µL cell lysis buffer per well (Abcam) on a PHMP Grant-Bio Thermoshaker. Absorbance at 570 nm was measured on a CLARIOStar plate reader (BMG Labtech), and cell viability was expressed as percentage of medium control.

## Supplementary Material

Appendix 01 (PDF)

## Data Availability

The data that support the findings of this study are available on Zenodo at 10.5281/zenodo.10059797.
