# Supplementary material for: Modulation of α-synuclein in vitro aggregation kinetics by its alternative splice isoforms
Source: Proc Natl Acad Sci U S A. 2024 Feb 7;121(7):e2313465121. doi: 10.1073/pnas.2313465121 (PMC10873642; doi:10.1073/pnas.2313465121)
Supplement: Supplementary file 1 — Appendix 01 (PDF) [file pnas.2313465121.sapp.pdf]

Supplementary Information

Supplementary Methods

SDS-PAGE

NuPAGE LDS Sample Buffer (4x) was diluted into protein samples in a 1:4 ratio and the mixture was heated to 95 °C for 10 min. Samples were run on a 4–12% Bis-Tris NuPAGE gel using 1x NuPAGE SDS MES Running Buffer with SeeBlue Plus2 Pre-Stained Protein Standard (ThermoFisher Scientific) as protein marker. Subsequently, gels were stained with Instant Blue Coomassie Protein Stain (Abcam) (DOI: dx.doi.org/10.17504/protocols.io.4r3l22er4l1y/v1).

FTIR

FTIR measurements were performed on a Vertex 70 FTIR spectrometer (Bruker) using a *DiamondATR* unit and a deuterated lanthanum α-alanine-doped triglycine sulphate detector (DOI: dx.doi.org/10.17504/protocols.io.dm6gp3xqpvzp/v1). αSyn aggregation reactions were performed as described for kinetic experiments, whereas no ThT was used in these samples. Fibrils were recovered after 6 days of incubation by centrifugation (20 min, 21100 *g*, RT), the pellet was washed once in MQ water, centrifuged again and resuspended in a final volume of 20 µL MQ water. 5 µL of protein solution were deposited on the prism and spectra were acquired over the spectral range of 4000–900 cm^{-1}. The spectra were then smoothened (25 points) and normalized. Due to the clumpy nature and therefore scattering effects of αSyn-112 and αSyn-98 aggregates, no FTIR spectra could be obtained at first. For comparison of the isoforms alone (**Figure S5A**), aggregates were therefore sonicated in an Ultrawave Ultra BT U100 water bath sonicator for 40 min, which did not affect the FTIR spectra comparing sonicated (**Figure S5A**) and unsonicated αSyn-140 fibrils (**Figure S5B,C**) but allowed the acquisition of FTIR spectra of αSyn-112 and αSyn-98.

Table S1. Kinetic parameters derived from monomer aggregation experiments.

	**αSyn-140**	**αSyn-126**	**αSyn-112**	**αSyn-98**
**k_+k_n [M^{-2} h^{-2}]**	$1.53x10^4$	$2.71x10^4$	$1.01x10^7$	$6.16x10^7$
**k_+k_- [M^{-1} h^{-2}]**	156	210	$8.87x10^3$	$4.54x10^4$
**K_E [M]**	$2.64x10^{-5}$	$5.08x10^{-4}$	$5.99x10^{-7}$	$2.27x10^{-7}$
**n_c**	2	2	2	2

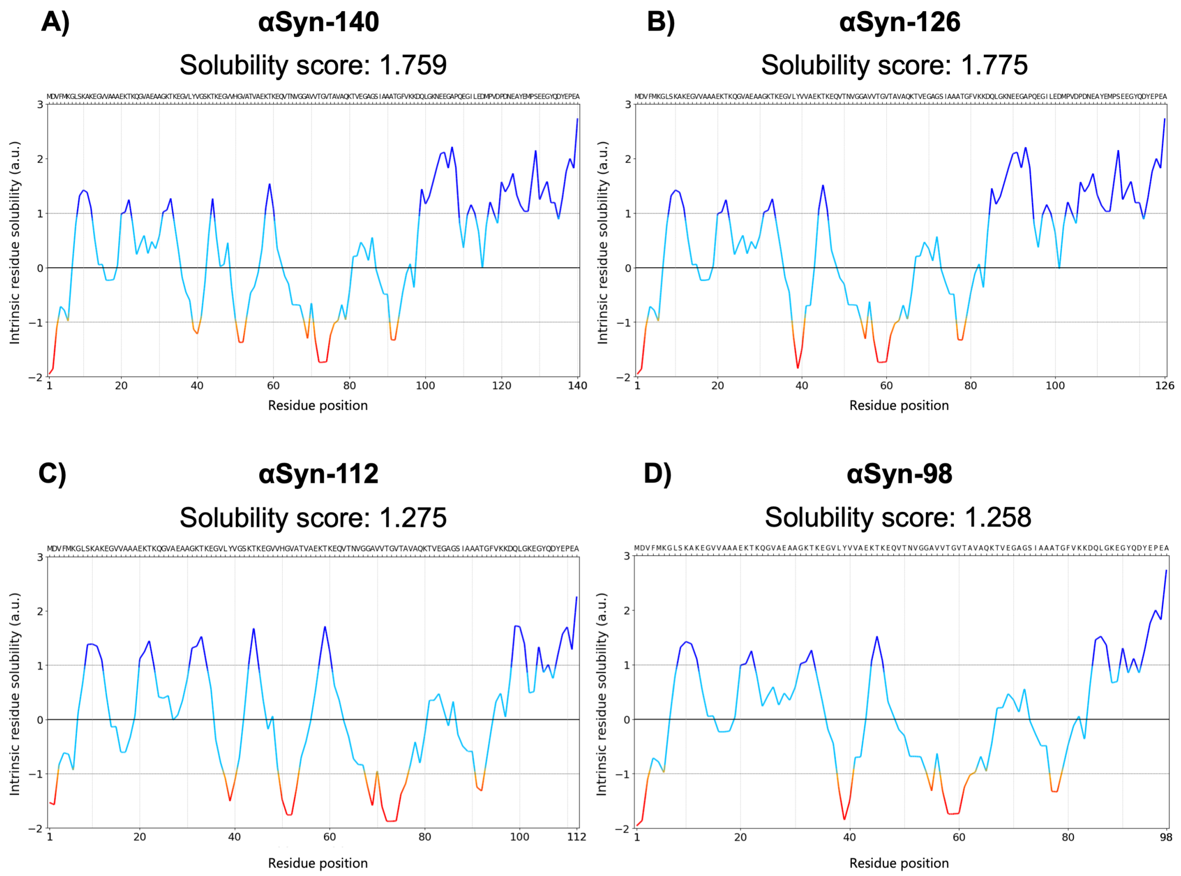

Figure S1. Predicted solubility profiles of the four αSyn splice isoforms analyzed in this work. (A–D) Overall solubility scores and residue-specific solubility profiles of αSyn-140 **(A)**, αSyn-126 **(B)**, αSyn-112 **(C)** and αSyn-98 **(D)** at pH 7.4; high values (blue) correspond to soluble regions, and low values (red) to insoluble regions. Due to the lack of the highly soluble region of residues 103–130, which is encoded by exon 5, the overall solubility scores are lower for αSyn-112 and αSyn-98 compared to αSyn-140 and αSyn-126.

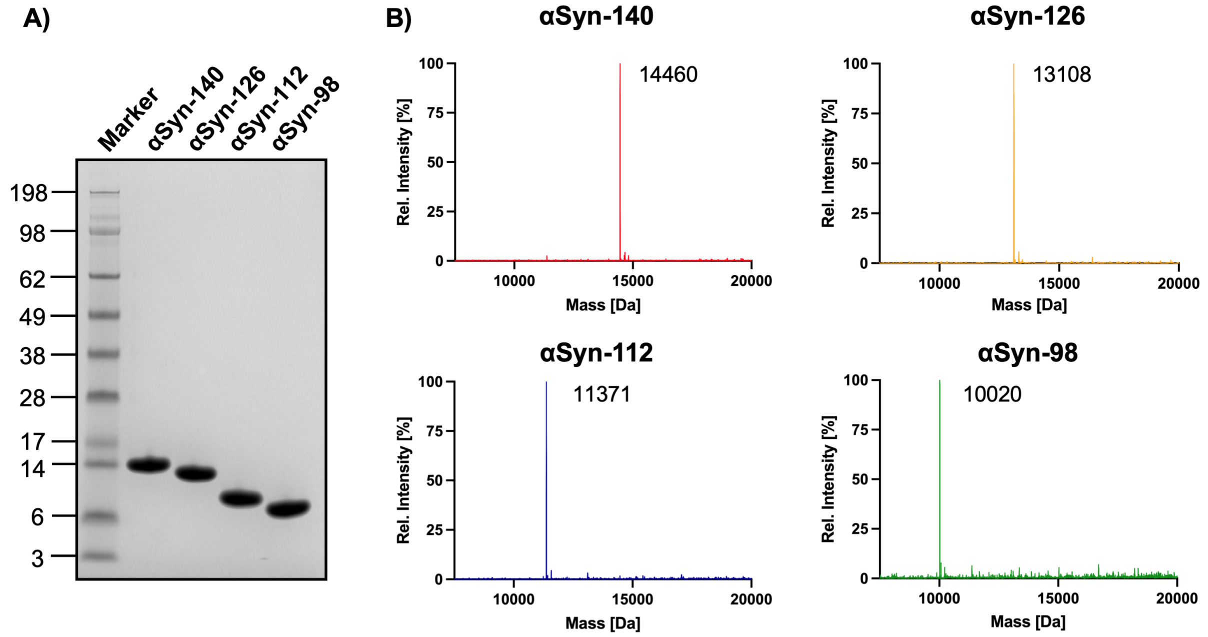

Figure S2. Quality assessment of purified αSyn isoforms. (**A**) Purity of the final protein product was assessed by SDS-PAGE followed by Coomassie stain. (**B**) The molecular mass of the produced αSyn isoforms was validated by LC-MS, as shown in the deconvoluted mass spectra.

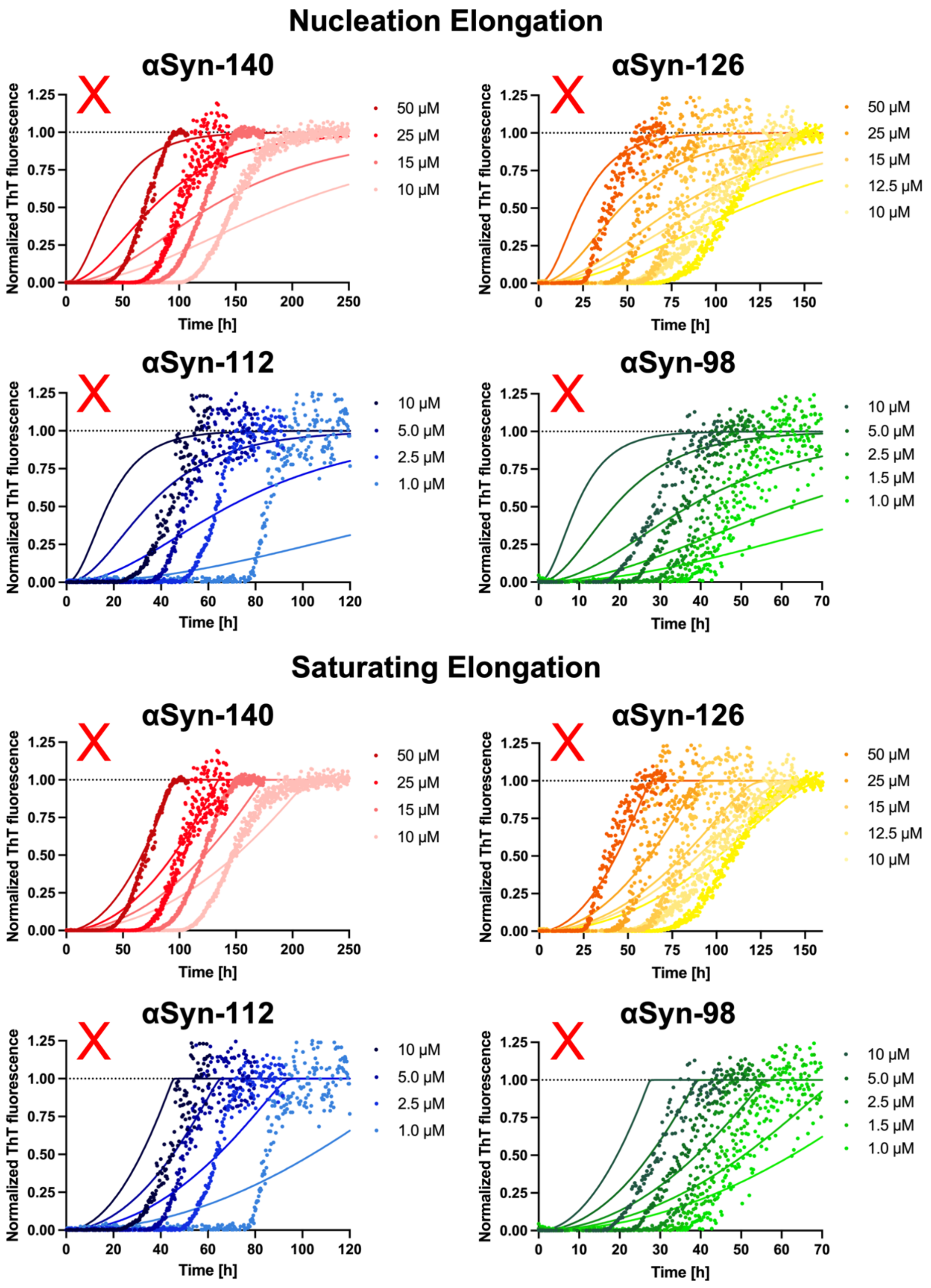

Figure S3. Misfits of αSyn isoform aggregation kinetics. Normalized traces (left) were fitted on the AmyloFit platform to the **(A)** *Nucleation Elongation* and **(B)** *Saturating Elongation* models, shown as the solid lines. These additional models are not in line with the scaling exponent, show a marked deviation from the data and are therefore less suitable than the *Saturating Elongation and Fragmentation* model chosen in **Figure 1**.

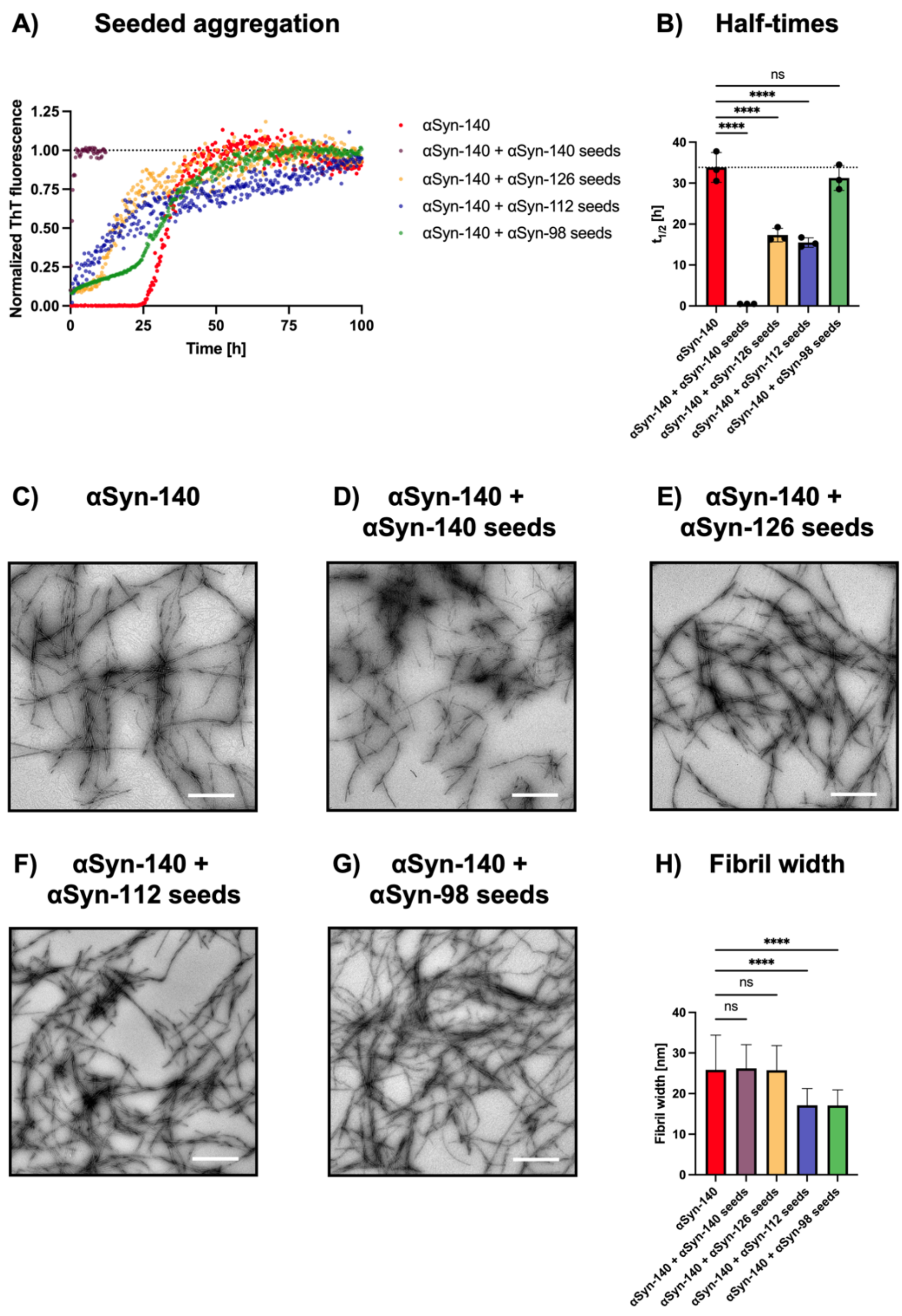

Figure S4. Seeded aggregation of αSyn-140. (**A**) Normalized aggregation kinetics of 90% (45 μM) αSyn-140 co-incubated with 10% (5 μM) fibril seeds derived from αSyn-140 (self-seeded), αSyn-126, αSyn-112, or αSyn-98 (cross-seeded), or 100% αSyn-140 (unseeded). While the αSyn-140 self-seeded reaction reached plateau rapidly, $t_{1/2}$ values of cross-seeded reactions were significantly increased for αSyn-126 seeds and αSyn-112 seeds, and highest for αSyn-98 seeds, which were similar to $t_{1/2}$ values of the unseeded reaction **(B)**. (**C–G**) Representative TEM images of amyloid fibrils formed by αSyn-140 unseeded, self-seeded and cross-seeded aggregation. **(H)** Image analysis revealed significantly lower fibril widths of αSyn-140 cross-seeded with αSyn-112 and αSyn-98 compared to αSyn-140 seeded with αSyn-140 and αSyn-126, and the αSyn-140 unseeded reaction. Data are shown as mean ± SD. One-way ANOVA using Dunnett's post-hoc test. ****$p<0.0001$, ns = non-significant, scale bars = 1 μm.

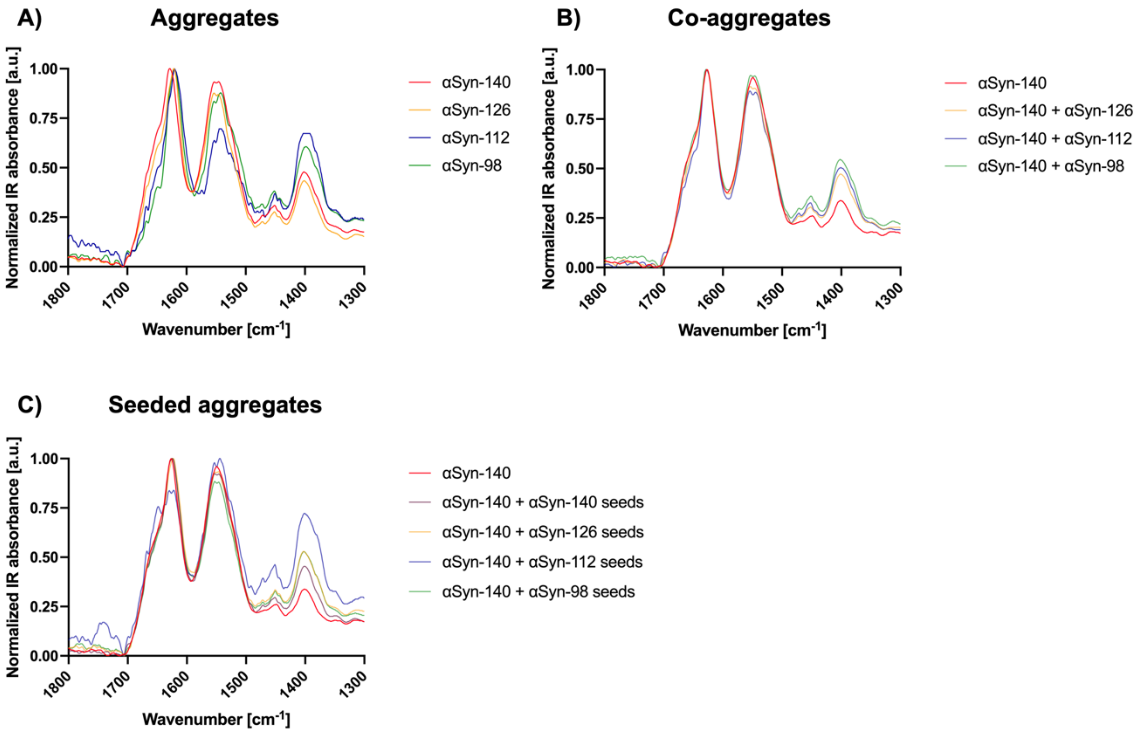

Figure S5. FTIR analysis of αSyn isoform aggregates. Spectra of fibrils formed by (**A**) aggregation of 100% αSyn-140, αSyn-126, αSyn-112 and αSyn-98, (**B**) co-aggregation of 90% αSyn-140 with 10% αSyn-126, αSyn-112 or αSyn-98 monomers compared to 100% αSyn-140, and (**C**) seeded aggregation of 90% αSyn-140 monomers with 10% αSyn-140, αSyn-126, αSyn-112 or αSyn-98 fibril seeds compared to 100% αSyn-140.

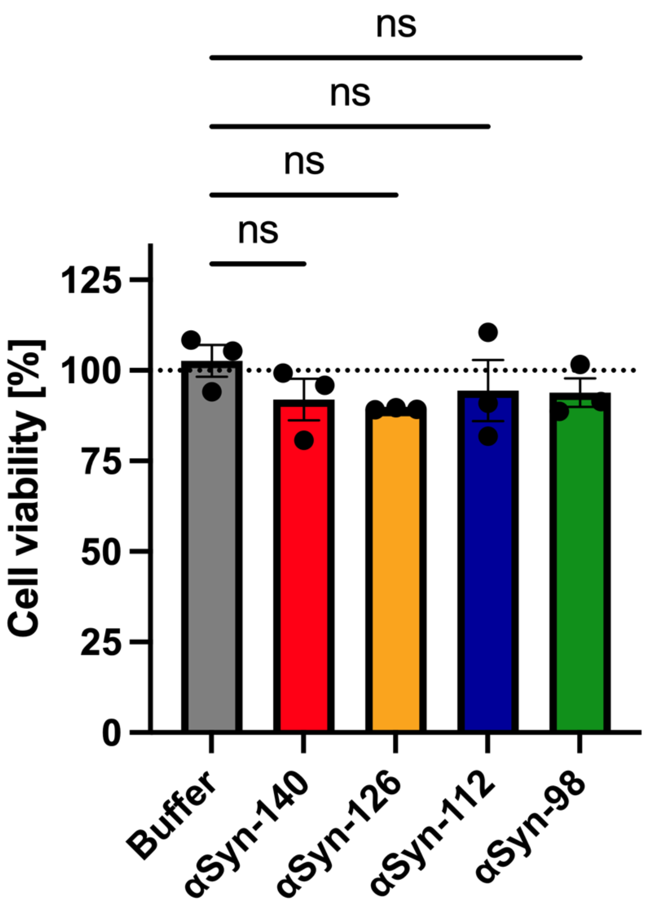

Figure S6. Monomers of αSyn isoforms are not cytotoxic to SH-SY5Y cells. Cell viability of SH-SY5Y cells was determined using an MTT assay after treatment with monomers of αSyn-140, αSyn-126, αSyn-112 and αSyn-98 at 50 µM. Data are expressed as percentage of medium control and shown as mean ± SEM of independent cell treatments (n = 3). One-way ANOVA with Dunnett's post-hoc test, ns = non-significant.
